# An Advanced Machine Learning Model for a Web-Based Artificial Intelligence–Based Clinical Decision Support System Application: Model Development and Validation Study

**DOI:** 10.2196/56022

**Published:** 2024-09-04

**Authors:** Tai-Han Lin, Hsing-Yi Chung, Ming-Jr Jian, Chih-Kai Chang, Cherng-Lih Perng, Guo-Shiou Liao, Jyh-Cherng Yu, Ming-Shen Dai, Cheng-Ping Yu, Hung-Sheng Shang

**Affiliations:** 1 Division of Clinical Pathology Department of Pathology Tri-Service General Hospital, National Defense Medical Center Taipei Taiwan; 2 Division of General Surgery Department of Surgery Tri-Service General Hospital, National Defense Medical Center Taipei Taiwan; 3 Division of Oncology Department of Internal Medicine Tri-Service General Hospital, National Defense Medical Center Taipei City Taiwan; 4 Department of Pathology Tri-Service General Hospital, National Defense Medical Center Taipei Taiwan

**Keywords:** breast cancer recurrence, artificial intelligence–based clinical decision support system, machine learning, personalized treatment planning, ChatGPT, predictive model accuracy

## Abstract

**Background:**

Breast cancer is a leading global health concern, necessitating advancements in recurrence prediction and management. The development of an artificial intelligence (AI)–based clinical decision support system (AI-CDSS) using ChatGPT addresses this need with the aim of enhancing both prediction accuracy and user accessibility.

**Objective:**

This study aims to develop and validate an advanced machine learning model for a web-based AI-CDSS application, leveraging the question-and-answer guidance capabilities of ChatGPT to enhance data preprocessing and model development, thereby improving the prediction of breast cancer recurrence.

**Methods:**

This study focused on developing an advanced machine learning model by leveraging data from the Tri-Service General Hospital breast cancer registry of 3577 patients (2004-2016). As a tertiary medical center, it accepts referrals from four branches—3 branches in the northern region and 1 branch on an offshore island in our country—that manage chronic diseases but refer complex surgical cases, including breast cancer, to the main center, enriching our study population’s diversity. Model training used patient data from 2004 to 2012, with subsequent validation using data from 2013 to 2016, ensuring comprehensive assessment and robustness of our predictive models. ChatGPT is integral to preprocessing and model development, aiding in hormone receptor categorization, age binning, and one-hot encoding. Techniques such as the synthetic minority oversampling technique address the imbalance of data sets. Various algorithms, including light gradient-boosting machine, gradient boosting, and extreme gradient boosting, were used, and their performance was evaluated using metrics such as the area under the curve, accuracy, sensitivity, and *F*_1_-score.

**Results:**

The light gradient-boosting machine model demonstrated superior performance, with an area under the curve of 0.80, followed closely by the gradient boosting and extreme gradient boosting models. The web interface of the AI-CDSS tool was effectively tested in clinical decision-making scenarios, proving its use in personalized treatment planning and patient involvement.

**Conclusions:**

The AI-CDSS tool, enhanced by ChatGPT, marks a significant advancement in breast cancer recurrence prediction, offering a more individualized and accessible approach for clinicians and patients. Although promising, further validation in diverse clinical settings is recommended to confirm its efficacy and expand its use.

## Introduction

Breast cancer, the most diagnosed cancer among women, presents a global health crisis with an extensive impact across diverse populations [1]. In 2022, more than 40,000 lives were taken due to breast cancer in the United States [2]. Characterized by its complexity and varied patient responses, breast cancer underscores the critical need for accurate prediction and management, particularly for recurrences [3]. This challenge necessitates the development of advanced personalized treatment strategies [3]. The effective prediction of recurrence significantly influences treatment decisions and patient outcomes [4].

Despite the numerous breast cancer prediction models, there is a notable gap in their practical applications [5]. Although scientifically sound, these models are often inaccessible to the broader medical community and patients [6]. This disconnection undermines their use in clinical settings, particularly in aiding treatment decisions [7]. The complexity of choosing an appropriate treatment strategy, even with guidelines, remains a challenge [3,7]. Guidelines are used to recommend the type of procedure, antiestrogen therapy, aromatase inhibitors, chemotherapy, and radiotherapy; however, the decision-making process using the guidelines can be daunting and complicated for both doctors and patients [3,4,7,8].

To bridge this gap, recent technological advancements have aimed to make predictive tools more accessible and user-friendly [9]. The move toward intuitive medical applications signifies a leap forward in the demystification of advanced tools for both clinicians and patients [9]. The introduction of artificial intelligence (AI) technologies, notably ChatGPT, promises to revolutionize this area [10]. With AI, the creation of efficient and intuitive predictive models has become increasingly feasible [11]. These advancements lay the groundwork for developing an AI-based clinical decision support system (AI-CDSS) that transforms the way clinicians and patients navigate their treatment choices [10].

The primary goal of this study was to use ChatGPT to develop a practical AI-CDSS tool for predicting breast cancer recurrence. This tool was designed to assist clinicians and patients in navigating the complex landscape of treatment decisions. By leveraging ChatGPT, the model simplifies the intricate data processes and feature selection, thereby enhancing the decision-making process regarding various therapy options. This study explores the development of this AI-enhanced tool, focusing on its application in clinical settings and its potential to significantly impact breast cancer management and patient care.

## Methods

### Research Design and Overview

This study focused on developing an advanced machine learning model to predict breast cancer recurrence. Central to this endeavor is the use of ChatGPT, which aims to enhance the model’s accuracy and make it user-friendly for clinical decision support. The hypothesis underpinning this research is that ChatGPT integration will not only improve the model’s effectiveness but also simplify its application, offering tangible benefits to medical professionals and patients in making informed treatment decisions.

### Ethical Considerations

This study was conducted in strict accordance with the ethical standards outlined in the Declaration of Helsinki and applicable local regulations for the protection of human subjects. It was approved by the institutional review board of Tri-Service General Hospital, Taipei, Taiwan (TSGHIRB B202005044). Given the retrospective nature of the research, the requirement for informed consent was waived. All data analyzed were anonymized to ensure confidentiality.

### Data Collection and Source Description

**Figure 1:**
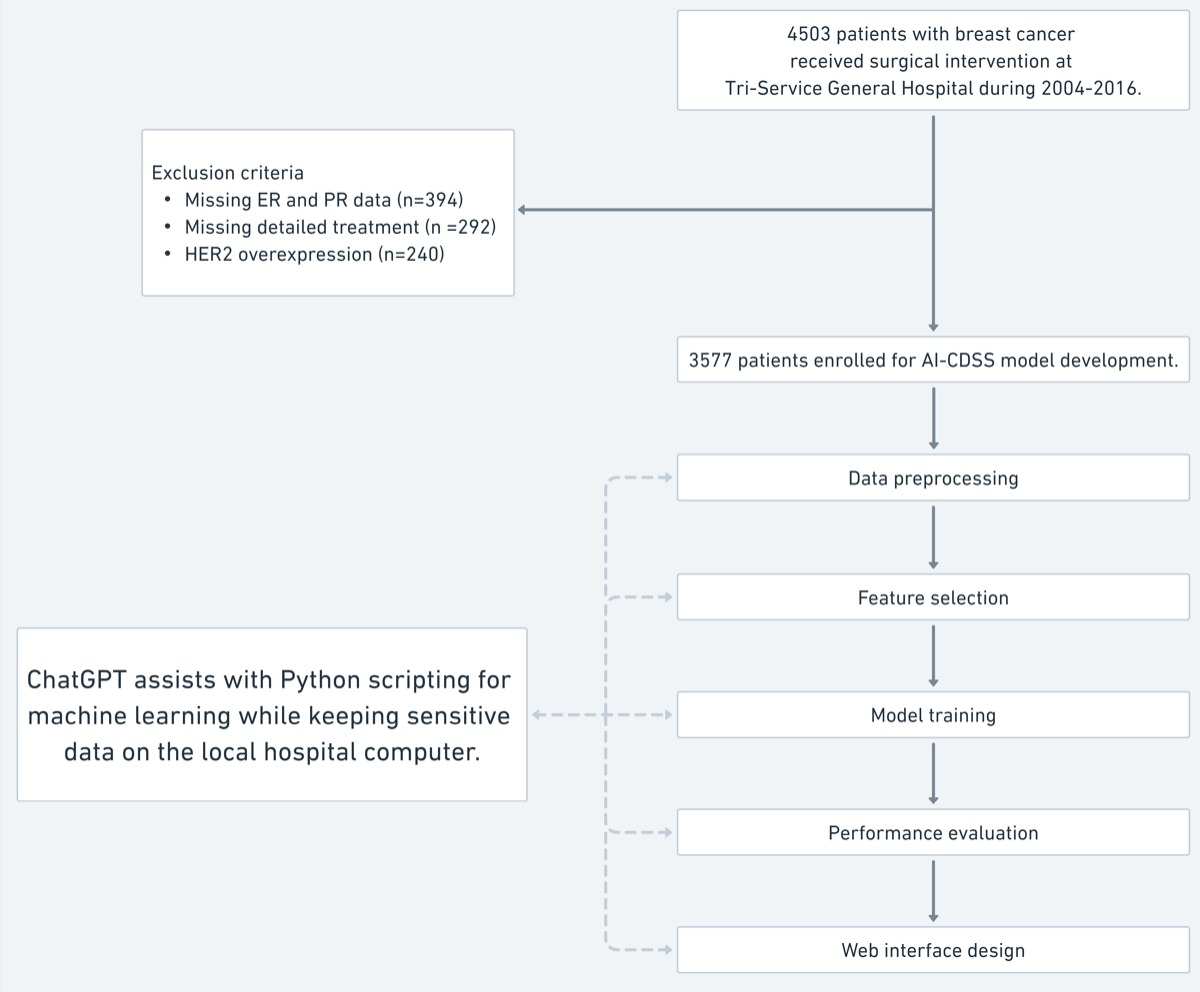
Patient selection flowchart and model development. This figure provides a flowchart outlining the process of developing the AI-CDSS model using data from the Tri-Service General Hospital breast cancer registry. It starts with the initial number of patients with breast cancer who received surgical intervention during 2004-2016 and follows through the stages of exclusion criteria application, data preprocessing, feature selection, model training, performance evaluation, and web interface design. The flowchart also notes the assistance of ChatGPT with Python scripting for machine learning while keeping sensitive data on the local hospital computer, highlighting the integration of AI in model development. AI: artificial intelligence; AI-CDSS: artificial intelligence–based clinical decision support system; ER: estrogen receptor; HER2: human epidermal growth factor receptor 2; PR: progesterone receptor.

This study used data from the Tri-Service General Hospital’s breast cancer registry in Taiwan, spanning from 2004 to 2016, with the study’s retrospective nature allowing the institutional review board (TSGHIRB B202005044) to waive the requirement for patient consent. The initial data set comprised 4503 patients who had undergone surgical treatment at our main medical center, which is part of a network, including 3 branches in the northern region and 1 branch on an offshore island in our country. These branches primarily manage chronic diseases but refer complex surgical cases, including breast cancer, to our main center, thus enriching the diversity of our study population across different health care settings. Essential parameters collected included age, estrogen receptor (ER) and progesterone receptor (PR) percentages and intensities, pathological stage, tumor size, nodal involvement, metastasis, and detailed treatment type, such as surgery type, antiestrogen therapy, aromatase inhibitor usage, chemotherapy, and radiotherapy. To refine the data set for AI-CDSS model development, exclusion criteria were applied: incomplete hormone receptor data (394 patients), missing detailed treatment records (292 patients), and human epidermal growth factor receptor 2 (HER2) overexpression (240 patients), focusing the study on non-HER2 mediated recurrence. These exclusions resulted in a final cohort of 3577 patients, as shown in Figure 1, ensuring data quality and homogeneity for effective analysis.

### Integration of ChatGPT in AI-CDSS Model Development

Scripting and data processing were conducted on secure, local hospital systems using Python (version 3.9, Python Software Foundation) in Jupyter Notebooks (Project Jupyter), with no external data transfers or cloud processing to ensure the confidentiality and security of patients. ChatGPT’s guidance was crucial in navigating the complexities of AI-CDSS development, enhancing task efficiency and accuracy on local systems. Below is a step-by-step breakdown of how ChatGPT supported each development process:

#### Data Preprocessing

ChatGPT assisted in crafting Python scripts for cleaning, normalizing numerical inputs, and encoding categorical variables, all of which were executed within the secure environment of local Jupyter Notebooks.

#### Feature Selection

Under ChatGPT’s guidance, we developed scripts to generate heatmaps for visualizing data correlations and distributions, which aided effective feature selection. For feature engineering, hormone receptors were categorized into detailed intervals, age was segmented by a 50-year threshold, and treatment types and tumor characteristics were appropriately formatted for further analysis.

#### Model Training, Validation, and Performance Evaluation

Model training and performance evaluation scripting were supported by ChatGPT. It assisted in setting and optimizing parameters for various machine-learning algorithms through Jupyter Notebooks on local hospital computers. ChatGPT helped develop scripts for evaluating model performance and obtaining detailed reports and visualizations, such as confusion matrices and receiver operating characteristic (ROC) curves, ensuring comprehensive and precise analysis.

#### AI-CDSS Web Interface Design

Guidance from ChatGPT was instrumental in developing scripts for a secure web interface that allows clinicians and patients to interact with the AI-CDSS efficiently by using local computing resources.

### Data Preprocessing

Data preprocessing was conducted using Python 3.9 in Jupyter Notebooks on secure local hospital systems, adhering to health care data protection regulations. We transformed the raw CSV data into Pandas DataFrames, enhancing data set manageability and facilitating accurate variable classification as categorical or continuous. ChatGPT helped create a function that appended a new column to the DataFrames to serve as the outcome variable for our models. This function calculated the time difference between the surgical resection date and any recorded recurrence date. If no recurrence was recorded or occurred more than 5 years post surgery, the outcome was classified as 0; otherwise, it was classified as 1.

### Feature Selection

**Figure 2:**
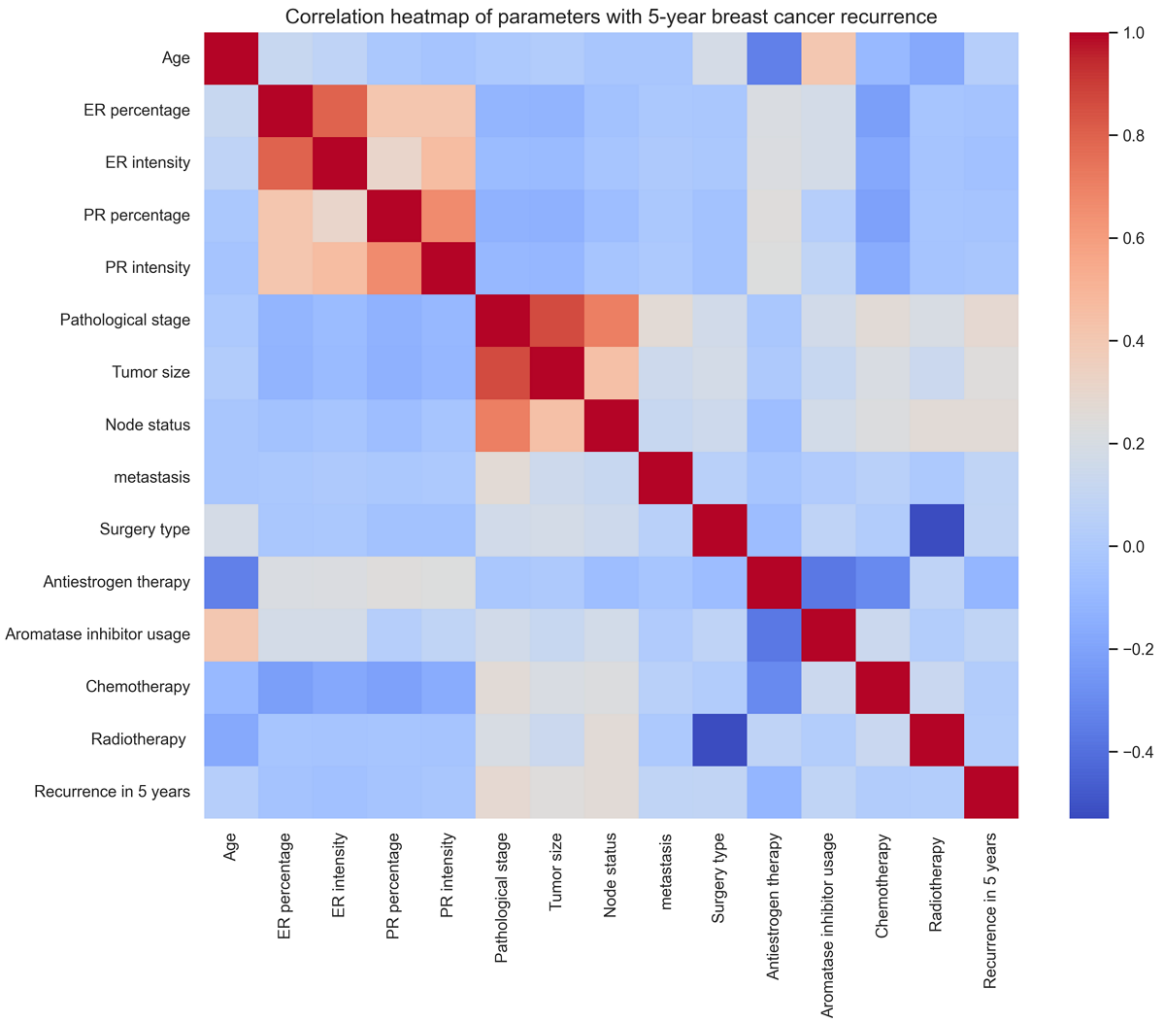
Correlation heatmap of parameters with 5-year breast cancer recurrence. The heat map depicts the correlations between various parameters and the occurrence of 5-year breast cancer recurrence. This effectively shows the relevance of each parameter in predicting breast cancer recurrence and serves as a crucial tool for identifying and selecting key features for the machine learning model. The color gradients in the heatmap correspond to the varying strengths of these correlations. ER: estrogen receptor; PR: progesterone receptor.

When developing our machine learning models, efficient feature selection was pivotal for optimizing performance. We developed Python scripts for generating and analyzing a heatmap, which significantly enhanced our understanding of the relationships between variables and 5-year breast cancer recurrence. The heatmap, shown in Figure 2, illustrates the correlations between various parameters such as ER percentage, PR percentage, surgical types, and treatment modalities with the occurrence of breast cancer recurrence within 5 years. The color gradients in the heatmap represent varying strengths of these correlations, providing a visual and quantitative method to identify and select the most predictive features for our models. This approach streamlined the feature selection process and ensured that our models were trained on variables most relevant to predicting early recurrence, enhancing their accuracy and clinical relevance.

After visualizing relationships between variables and 5-year breast cancer recurrence through heatmaps, we used feature engineering for optimal data preparation for machine learning analysis. This process involved the intricate categorization and transformation of key variables. ER and PR receptor percentages were divided into negative and positive groups, with the latter further divided into 10 incremental categories at 10% intervals, enhancing the granularity for recurrence outcome prediction. Age was categorized into 2 groups using a 50-year threshold to differentiate risk levels associated with younger and older age groups. We applied one-hot encoding to categorical data, including treatment types and tumor characteristics, ensuring all variables were accurately represented in a machine-readable format. These feature engineering steps were crucial for developing robust predictive models, enabling the models to effectively use the most relevant and precisely formatted data.

### Model Training, Validation, and Performance Evaluation

Model training used patient data in 2004-2012, with subsequent validation using data in 2013-2016, ensuring comprehensive assessment and robustness of our predictive models. Under ChatGPT’s guidance, we selected a range of advanced machine learning algorithms to effectively tackle the complexities inherent in breast cancer recurrence data. The algorithms chosen included light gradient boosting machine (LGBM), gradient boosting (GB), extreme gradient boosting (XGB), random forest (RF), AdaBoost, and artificial neural networks, each selected for their unique strengths in addressing different aspects of the data set [12,13]. LGBM is a highly efficient and scalable gradient-boosting framework optimized for fast training, low memory usage, and high accuracy, capable of handling large data sets. GB is a powerful machine learning technique that combines multiple weak models, sequentially correcting their errors to improve accuracy but requires significant computational resources. XGB is an efficient, high-accuracy machine learning algorithm with early stopping and built-in regularization to prevent overfitting, well-suited for large data sets. RF is an ensemble machine learning model that builds multiple decision trees using random subsets of features and data to improve accuracy and reduce overfitting, effectively handling classification tasks. AdaBoost is an ensemble algorithm that sequentially trains weak classifiers, adjusting for errors by reweighting instances to improve accuracy and combining their weighted predictions for robust classification. Artificial neural networks are brain-inspired computational models with interconnected layers that effectively learn complex patterns from large data sets, suitable for classification tasks but require significant computational power and resources.

To assess the effectiveness of our machine learning models, we used several key performance metrics such as area under the curve (AUC), accuracy, sensitivity, specificity, positive predictive value (PPV), negative predictive value (NPV), and *F*_1_-score. We implemented these metrics using the *scikit-learn* Python library, specifically using functions, such as roc_auc_score, accuracy_score, recall_score, precision_score, and f1_score from the sklearn.metrics module. This approach was crucial for estimating general accuracy and evaluating how effectively the models identified true positives and negatives, an essential factor in the clinical context of predicting breast cancer recurrence.

Due to a significant disparity in the number of cases between groups with and without recurrence, we used the synthetic minority oversampling technique (SMOTE) to balance the class distribution. This strategy enhanced the overall accuracy and generalizability of our predictive models. By mitigating bias toward the larger group of patients without recurrence, we were able to ensure with this proactive approach that our models could learn more effectively from a more equitably distributed data set. After correcting class imbalances with SMOTE and selecting appropriate algorithms, we conducted extensive hyperparameter tuning. This process involved systematically testing various combinations of hyperparameter values on local hospital computers to identify the optimal settings for each model. These settings are shown in Multimedia Appendix 1.

### AI-CDSS Web Interface Design

After identifying optimal parameters for various advanced machine learning algorithms, we saved the best-performing models using the *joblib* library. This enabled seamless integration into a web-based interface, enhancing patient care and supporting clinicians in making informed decisions. With assistance from ChatGPT, we developed a web page built with HTML, CSS, and JavaScript, featuring dynamic forms where clinicians could input patient data and receive immediate predictions on breast cancer recurrence. The backend, powered by a Flask server, uses these serialized models to compute predictions based on user input. This setup not only ensures that the interface is a practical tool for clinical decision support but also aligns with the transformative goals of the AI-CDSS tool in managing breast cancer.

Furthermore, we provide ongoing support through webinars and one-on-one training sessions to accommodate different learning preferences, ensuring users feel confident using the AI-CDSS tool in their clinical practice. We collect regular feedback from users through surveys and interviews to continuously improve the tool and address emerging needs. By offering extensive training and support, we aim to maximize the tool’s usability and integration into everyday clinical workflows, ultimately enhancing patient care.

## Results

### Characteristics of the Study Population

The data set, derived from the Tri-Service General Hospital breast cancer registry, encompassed 3577 patients treated between 2004 and 2016. Demographic and clinical variables such as hormone receptor status, tumor characteristics, and treatment modalities were meticulously collected. Most patients were older than 50 years of age, and the majority were ER and PR positives. A significant proportion of patients underwent conservative surgery, and adjuvant therapies such as chemotherapy, antiestrogen therapy, and radiotherapy were commonly administered (Table 1).

**Table 1:** Patient demographics, tumor characteristics, and treatment details.

						Overall (N=3577)			Training (n=2503)			Validation (n=1074)
**Recurrence within 5 years, n (%)**												
			No		3152 (88.1)			2208 (88.2)			944 (87.9)	
			Yes		425 (11.9)			295 (11.8)			130 (12.1)	
Age (years), mean (SD)						52.97 (11.29)			52.99 (11.18)			52.92 (11.55)
**Estrogen receptor status, n (%)**												
			Negative		799 (22.3)			569 (22.7)			230 (21.4)	
			Positive		2778 (77.7)			1934 (77.3)			844 (78.6)	
**Progesterone receptor status, n (%)**												
			Negative		658 (18.4)			470 (18.8)			188 (17.5)	
			Positive		2919 (81.6)			2033 (81.2)			886 (82.5)	
**Estrogen receptor intensity, n (%)**												
			0		799 (22.3)			569 (22.7)			230 (21.4)	
			1		216 (6.0)			140 (5.6)			76 (7.1)	
			2		1789 (50.01)			1263 (50.5)			526 (48.98)	
			3		773 (21.6)			531 (21.2)			242 (22.5)	
**Progesterone receptor intensity, n (%)**												
			0		656 (18.3)			468 (18.7)			188 (17.5)	
			1		492 (13.8)			341 (13.6)			151 (14.1)	
			2		1887 (52.8)			1327 (53.01)			560 (52.1)	
			3		542 (15.2)			367 (14.7)			175 (16.3)	
**Pathological stage, n (%)**												
			Stage 0		997 (27.9)			707 (28.2%)			290 (27.0)	
			Stage 1		1097 (30.7)			750 (30.0)			347 (32.3)	
			Stage 2		1036 (28.96)			731 (29.2%)			305 (28.4)	
			Stage 3		395 (11.04)			278 (11.1)			117 (10.9)	
			Stage 4		52 (1.5)			37 (1.5)			15 (1.4)	
**Tumor size, n (%)**												
			T0		1003 (28.04)			711 (28.4)			292 (27.2)	
			T1		1432 (40.03)			985 (39.4)			447 (41.6)	
			T2		964 (26.9)			683 (27.3)			281 (26.2)	
			T3		122 (3.4)			86 (3.4)			36 (3.4)	
			T4		56 (1.6)			38 (1.5)			18 (1.7)	
**Nodal involvement, n (%)**												
			N0		2646 (73.97)			1834 (73.3)			812 (75.6)	
			N1		574 (16.04)			419 (16.7)			155 (14.4)	
			N2		231 (6.5)			162 (6.5)			69 (6.4)	
			N3		126 (3.5)			88 (3.5)			38 (3.5)	
**Metastasis, n (%)**												
			M0		3541 (98.99)			2475 (98.9)			1066 (99.3)	
			M1		36 (1.01)			28 (1.1)			8 (0.7)	
**Treatment details**												
	**Surgery type**											
		Breast-conserving surgery		1363 (38.1)			966 (38.6)			397 (36.96)		
		Modified radical mastectomy		2214 (61.9)			1537 (61.4)			677 (63.03)		
	**Antiestrogen therapy**											
		No		1382 (38.6)			978 (39.1)			404 (37.6)		
		Yes		2195 (61.4)			1525 (60.9)			670 (62.4)		
	**Aromatase inhibitor usage**											
		No		2314 (64.7)			1608 (64.2)			706 (65.7)		
		Yes		1263 (35.3)			895 (35.8)			368 (34.3)		
	**Chemotherapy**											
		No		897 (25.1)			607 (24.3)			290 (27)		
		Yes		2680 (74.9)			1896 (75.7)			784 (73.0)		
	**Radiotherapy**											
		No		1910 (53.4)			1326 (52.98)			584 (54.4)		
		Yes		1667 (46.6)			1177 (47.02)			490 (45.6)		

### Model Performance Metrics, Validation Results, and ROC Curve Analysis

To ensure robustness, these models were validated using a separate validation data set comprising 1074 patients, with data from 2013 to 2016. After evaluating the performance of machine learning models using a suite of metrics, the LGBM model stood out, with an AUC of 0.80, demonstrating its robust predictive power in identifying breast cancer recurrence. The GB model followed closely with an AUC of 0.78, and the XGB model matched this performance, both reflecting a strong predictive capability. These models were further assessed for accuracy, sensitivity, specificity, PPV, NPV, and *F*_1_-score. Notably, LGBM achieved an accuracy of 0.88, sensitivity of 0.33, specificity of 0.96, PPV of 0.51, NPV of 0.91, and an *F*_1_-score of 0.40, underscoring a well-rounded performance.

**Figure 3:**
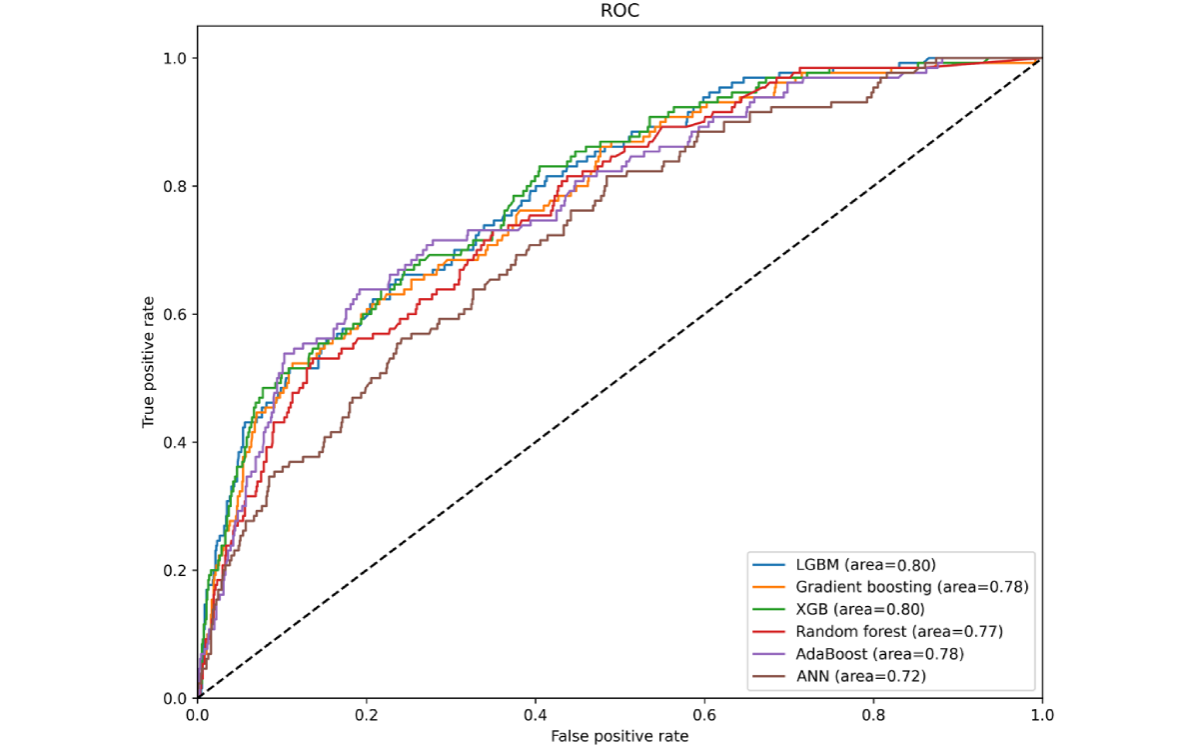
ROC curves of the evaluated machine learning models. This figure shows the ROC curves for the various machine learning models assessed in our study, including the LGBM, gradient boosting, XGB, random forest, AdaBoost, and ANN. These curves graphically represent the true positive rate versus the false positive rate for each model, elucidating their efficacy in differentiating recurrence from non-recurrence cases. The area under the curve values were also provided to gauge the predictive accuracy of each model. ANN: artificial neural networks; LGBM: light gradient boosting machine; ROC: receiver operating characteristic; XGB: extreme gradient boosting.

The diagnostic proficiency of the models was also analyzed using the ROC curve analysis. This method provides a visual comparison of each model’s true positive rate and false positive rate (Figure 3). The AUC obtained from the ROC curves reinforced the quantitative findings, illustrating that the LGBM, GB, and XGB models possess superior capabilities for discriminating between patients with and without breast cancer recurrence. The results from the ROC analysis and model metrics are shown in Table 2.

**Table 2:** Overview of machine learning models’ performance.

Model	Training AUC^a^	Testing AUC	Accuracy	Sensitivity	Specificity	PPV^b^	NPV^c^	*F*_1_-score
LGBM^d^	0.98	0.80	0.88	0.33	0.96	0.51	0.91	0.40
GB^e^	0.98	0.78	0.88	0.35	0.95	0.49	0.91	0.41
XGB^f^	0.98	0.80	0.88	0.39	0.94	0.49	0.92	0.44
RF^g^	0.99	0.77	0.87	0.24	0.95	0.42	0.90	0.30
AdaBoost	0.97	0.78	0.87	0.25	0.96	0.46	0.90	0.32
ANN^h^	0.99	0.72	0.84	0.22	0.93	0.30	0.90	0.25

^a^AUC: area under the curve.

^b^PPV: positive predictive value.

^c^NPV: negative predictive value.

^d^LGMB: light gradient boosting machine.

^e^GB: gradient boosting.

^f^XGB: extreme gradient boosting.

^g^RF: random forest.

^h^ANN: artificial neural network.

### User Interface Effectiveness and Case Studies

**Figure 4:**
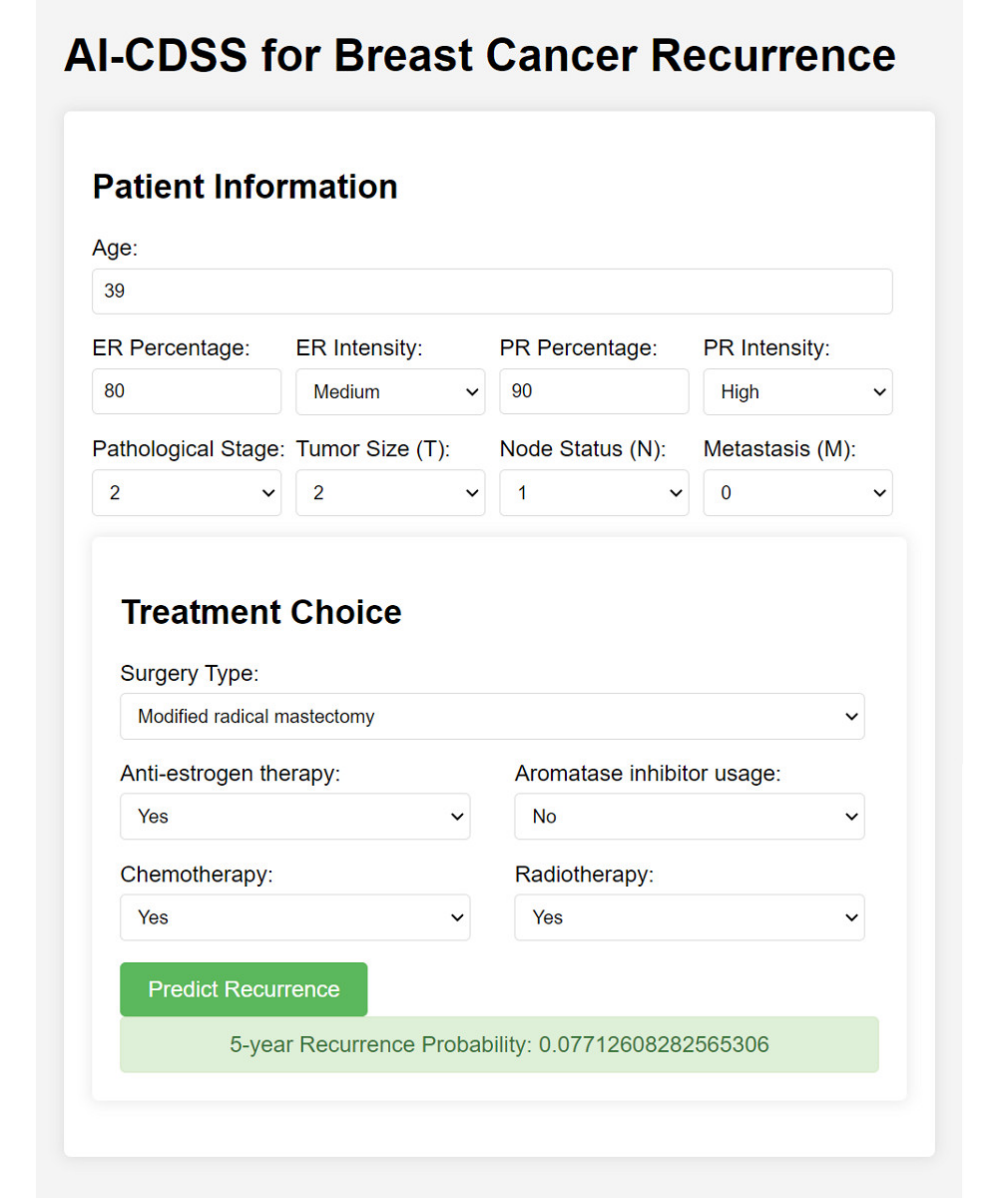
User interface of the AI-CDSS tool. This figure displays the web-based user interface of the AI-CDSS for breast cancer recurrence, showcasing the input fields for patient information, including age, hormone receptor percentages and intensities, pathological stage, tumor size, node status, and metastasis. It also details the treatment choices such as surgery type, antiestrogen therapy, aromatase inhibitor usage, chemotherapy, and radiotherapy. The interface features a “Predict Recurrence” button that, when clicked, calculates and displays the 5-year recurrence probability, demonstrating the tool’s capability for real-time, personalized recurrence risk assessment. AI-CDSS: artificial intelligence–based clinical decision support system.

The web interface of the AI-CDSS tool, depicted in Figure 4, was assessed for its efficacy in clinical decision-making. It offers a dynamic platform for clinicians and patients to enter critical patient data, such as age, hormone receptor status, and tumor characteristics, and explore various treatment options, including surgery, antiestrogen therapy, and radiotherapy. This web-based system enables the calculation of 5-year recurrence risks for different treatment choices, facilitating more informed and personalized decision-making. Its practicality in real-world scenarios has been underscored by case studies demonstrating its significant role in enhancing patient involvement and aiding clinicians in selecting appropriate treatment strategies.

To evaluate the integration of the AI-CDSS tool into clinical workflows, we use surveys and interviews to comprehensively assess the tool from multiple dimensions. The survey assesses usability aspects such as ease of use, navigation, and accessibility throughout patient care stages. A key focus is on the tool’s impact on decision-making, including how it influences treatment planning, personalizes options, and potentially improves outcomes. The survey also covers training adequacy, overall satisfaction, and the likelihood of recommendation. Open-ended questions are included to gather feedback on improvements and observations for a holistic perspective. The interviews align with the survey concepts, offering qualitative insights that complement the structured data. This approach ensures a comprehensive assessment of usability, integration, effectiveness, decision support capabilities, training quality, and user satisfaction from the users’ perspectives.

## Discussion

### Principal Results

The research marked a significant advancement in breast cancer management with the development of an AI-CDSS leveraging the capabilities of ChatGPT. This study used a comprehensive data set of 3577 patients from Tri-Service General Hospital, Taiwan. Key achievements include the meticulous categorization of hormone receptor status and age, which significantly enhances the precision of the data set for machine learning applications. Among the various models tested, the LGBM model showed superior performance, with an AUC of 0.80, a finding that was consistently supported by the ROC curve analysis. These results underscore the proficiency of the AI-CDSS tool for predicting breast cancer recurrence, thereby establishing a new benchmark in the field.

### Interpretations

The reliability of the LGBM model in predicting breast cancer recurrence reflects a broader trend in personalized medicine [14,15]. Compared to traditional models, our AI-CDSS tool provides a more tailored prediction, aligning with the current shift in oncological research toward individualized patient care [14,16]. The refined categorization of hormone receptor status and age groups in our model concurred with the findings of other studies, emphasizing the heterogeneity of breast cancer [14-16]. However, our model distinguishes itself by leveraging AI to address these complexities more effectively.

### Optimizing Model Selection for Clinical Contexts

In evaluating the optimal model to predict recurrence, each exhibited unique strengths and limitations suitable for specific clinical contexts. LGBM, GB, and XGB all showed a high accuracy of 0.88. LGBM excelled in rapid computation and achieved a highest AUC of 0.80, indicating a good balance between sensitivity (0.33) and specificity (0.96). GB offered high accuracy (0.88) and slightly better sensitivity (0.35), but its high computational demands limit its real-time practicality. XGB matched LGBM in AUC (0.80) and demonstrated the highest sensitivity (0.39) but required significantly more computational resources. Therefore, LGBM is preferable for rapid analyses prioritizing speed and balanced efficiency, while GB and XGB are ideal for resource-rich settings [12]. Selecting the appropriate model for each clinical context ensures optimal performance and reliability aligned with specific needs and resource constraints. If the clinical condition prioritizes early detection and aggressive treatment of recurrence, such as in more advanced stages of cancer, the sensitivity might be prioritized, making a model such as XGB preferred despite its higher computational requirements. However, if avoiding overtreatment and reducing patient anxiety are paramount, such as in early-stage cancer cases, which constituted 87.6% (N=3130) of the cases in our study (stages 0-2), specificity might be prioritized, making LGBM (with a specificity of 0.96) a better choice.

### Implications

Current trends in breast cancer research value precision and accessibility [14,17]. Unlike existing models that primarily emphasize scientific robustness, our AI-CDSS tool uniquely bridges the gap between high-level scientific accuracy and user-friendliness [17]. This balance is pivotal in the current era of breast cancer management, which is witnessing a shift toward greater patient involvement and clinician accessibility to advanced diagnostic tools. By offering a tool that is both technically sound and easily navigable, our study responds to the growing demand for personalized care and empowers health care providers with practical solutions [16,18]. Thus, the AI-CDSS tool stands out in the landscape of breast cancer predictive models, offering a nuanced and patient-centric approach that aligns with the evolving needs and expectations of patients with cancer and medical practitioners [18].

### Limitations

Although our findings were promising, this study had some limitations. Despite a robust initial data set of 4503 patients who had undergone surgical treatment at our main medical center, the study’s retrospective design and focus on data primarily from a single center pose inherent limitations. Although this center is supported by branches that refer to complex surgical cases, including breast cancer, the final cohort used for analysis was relatively small (N=3577), and the exclusion of certain patient groups (such as those with HER2 overexpression) may have impacted the generalizability of our results. To ensure fairness and transparency, we used SMOTE to balance the data set and selected machine learning algorithms known for their accuracy and interpretability. All data processing was conducted on secure local systems to protect patient privacy, with the AI-CDSS tool designed to assist, not replace, clinical judgment. Notably, 87.6% of our cases were early-stage cancer (stages 0-2), making the LGBM model, with a specificity of 0.96, a suitable core model to avoid overtreatment and reduce patient anxiety. However, varying patient demographics across different institutions may necessitate the adoption of different models. Continuous surveys and interviews are essential to comprehensively evaluate user needs. Future studies should include a more diverse patient cohort and consider real-time data to enhance the applicability and robustness of this model.

### Recommendations

We recommend a broader validation of the AI-CDSS tool across diverse clinical environments and patient populations to ascertain its efficacy and adaptability. Enriching the data set with a wider array of breast cancer cases would further improve the predictive accuracy of the model. Integrating patient feedback into the AI-CDSS interface could also enhance user-friendliness, potentially increasing its adoption and impact in clinical settings. Such efforts will not only validate our current findings but also pave the way for more advanced patient-centric cancer care solutions.

### Conclusions

In this research, we developed a web-based AI-CDSS application, enhanced by ChatGPT’s guidance, for predicting breast cancer recurrence. Using data from 3577 patients with breast cancer at Tri-Service General Hospital (2004-2016), the study applied advanced machine learning algorithms, including LGBM, GB, and XGB. ChatGPT significantly aided in data preprocessing, such as hormone receptor categorization, age binning, and one-hot encoding, and tackled data imbalance with SMOTE techniques. The models’ performances were evaluated using AUC, accuracy, sensitivity, and *F*_1_-score, with LGBM achieving the highest AUC of 0.80. The AI-CDSS tool’s web interface proved effective in clinical decision-making, enhancing personalized treatment plans and patient engagement. This study underscores AI-CDSS’s role, especially with ChatGPT’s contribution, in advancing personalized medicine and integrating health care technology, offering a more tailored approach to breast cancer recurrence prediction.

## Appendix

Multimedia Appendix 1 Parameters of machine learning models for predicting 5-year breast cancer recurrence.

## Abbreviations

AI: artificial intelligence
AI-CDSS: artificial intelligence–based clinical decision support system
AUC: area under the curve
ER: estrogen receptor
GB: gradient boosting
HER2: human epidermal growth factor receptor 2
LGBM: light gradient-boosting machine
NPV: negative predictive value
PPV: positive predictive value
PR: progesterone receptor
RF: random forest
ROC: receiver operating characteristic
SMOTE: synthetic minority oversampling technique
XGB: extreme gradient boosting
