# Supplementary material for: An Advanced Machine Learning Model for a Web-Based Artificial Intelligence–Based Clinical Decision Support System Application: Model Development and Validation Study
Source: J Med Internet Res. 2024 Sep 4;26:e56022. doi: 10.2196/56022 (PMC11411218; doi:10.2196/56022)
Supplement: Multimedia Appendix 1 [file jmir_v26i1e56022_app1.docx]

**Table S1.** Parameters of machine learning models for predicting 5-year breast cancer recurrence.

| **Model** | **Parameter** |  |
| --- | --- | --- |
| **LGBM** | n_estimators | 100 |
|  | max_depth | 7 |
|  | learning_rate | 0.05 |
|  | num_leaves | 50 |
|  | subsample | 0.8 |
|  | min_child_samples | 20 |
|  | colsample_bytree | 0.9 |
| **GB** | n_estimators | 300 |
|  | max_depth | 7 |
|  | learning_rate | 0.01 |
|  | subsample | 0.8 |
| **XGB** | n_estimators | 300 |
|  | max_depth | 7 |
|  | learning_rate | 0.01 |
|  | colsample_bytree | 0.8 |
|  | subsample | 0.9 |
| **RF** | n_estimators | 100 |
|  | max_depth | 20 |
|  | min_samples_split | 2 |
|  | min_samples_leaf | 1 |
| **AdaBoost** | n_estimators | 200 |
|  | learning_rate | 1 |
| **ANN** | activation | relu |
|  | alpha | 0.01 |
|  | learning_rate | constant |
|  | hidden_layer_sizes | (100, 50) |
|  | solver | adam |

Abbreviations: ANN, Artificial Neural Networks; GB, Gradient Boosting; LGMB, Light Gradient Boosting Machine; RF, Random Forest; XGB, Extreme Gradient Boosting.
